# Associations of Longitudinal Multiparametric MRI Findings and Clinical Outcomes in Intra-Articular Injections for Knee Osteoarthritis

**DOI:** 10.3390/diagnostics14182025

**Published:** 2024-09-13

**Authors:** Woo Young Kang, Suk-Joo Hong, Ji-Hoon Bae, Zepa Yang, In Seong Kim, Ok Hee Woo

**Affiliations:** 1 Department of Radiology, Korea University Guro Hospital, Seoul 08308, Republic of Korea; quartet0@hanmail.net (W.Y.K.); hongsj@korea.ac.kr (S.-J.H.); yangzepa@gmail.com (Z.Y.); 2 Department of Orthopedic Surgery, Korea University Guro Hospital, Seoul 08308, Republic of Korea; osman@korea.ac.kr; 3 Siemens Healthineers Ltd., Seoul 06620, Republic of Korea; inseong.kim@siemens-healthineers.com

**Keywords:** osteoarthritis, cartilage, magnetic resonance imaging

## Abstract

Background: Osteoarthritis (OA) is a complex disease marked by the degradation of articular cartilage. Objective: This study aimed to explore the relationship between cartilage volume/thickness and clinical outcomes in knee OA patients treated with intra-articular injections over one year. Methods: Twenty-four patients with mild-to-moderate OA were retrospectively analyzed using knee MRI. OA features were assessed semiquantitatively with the Whole-Organ Magnetic Resonance Imaging Score (WORMS), while cartilage thickness and volume in the medial femoral condyle (MFC) and medial tibial plateau (MTP) were measured. T1ρ and T2 values for MFC cartilage were also recorded. Clinical outcomes were evaluated using the Korean Western Ontario and McMaster Universities (K-WOMAC) and Knee Injury Osteoarthritis Outcomes (KOOS) scores. Spearman’s rank test assessed the associations between imaging changes and clinical outcomes. Results: The baseline MTP and MFC cartilage thickness and MTP cartilage volume showed significant correlations with clinical outcomes. Additionally, less progressive cartilage loss in the medial femorotibial joint (MFTJ) and overall joint was linked to a better clinical response over 12 months. Conclusions: In conclusion, thicker baseline MFTJ cartilage and minimal cartilage loss were associated with favorable clinical outcomes in knee OA patients receiving intra-articular injections.

## 1. Introduction

Osteoarthritis (OA) is the most prevalent chronic arthritis and is defined as a progressive disease of synovial joints due to failed repair of joint damage caused by stresses arising from an abnormality in any of the synovial joint tissues. Although cartilage degradation is the major hallmark of OA, recent studies have suggested that OA is a complex heterogeneous disease with different clinical and biochemical phenotypes involving all tissues of the joint [1,2].

Although the role of imaging in OA diagnosis and follow-up has not been clearly defined in clinical practice, it remains an essential tool for assessing disease progression and monitoring therapeutic response. Radiography is widely used for the diagnosis and monitoring of OA, typically focusing on identifying bone changes and joint space narrowing (JSN), which are evaluated using the Kellgren–Lawrence (KL) score [3]. The Federal Drug Administration (FDA) and European Medicines Agency (EMA) recommend radiographic JSN as the imaging endpoint for clinical trials of disease-modifying OA drugs (DMOADs). However, this approach has significant limitations. Radiography is less sensitive to early cartilage injuries, often fails to detect focal cartilage loss, and shows a poor correlation between joint structural pathology and symptomatic disease. Additionally, using JSN as the endpoint in clinical trials necessitates a large sample size and a follow-up period of 2–3 years to demonstrate the significant benefit of a novel therapy, making the process prohibitively expensive [4]. These challenges have dampened enthusiasm for the development of new therapeutic agents, underscoring the need for more sensitive and cost-effective imaging methods in OA research and drug development.

Magnetic resonance imaging (MRI) has expanded our comprehensive understanding of the pathogenesis of OA by allowing the assessment of cartilage morphologic features, biochemical composition, and other joint tissues contributing to the disease process [5]. Semiquantitative and quantitative assessments of cartilage on MRI are recommended to evaluate disease progression as an endpoint in clinical trials [6,7]. These MRI techniques have demonstrated adequate reliability, specificity, and sensitivity, along with the ability to detect lesion progression over a 1–2 year period [8].

Although numerous studies have demonstrated that imaging features are related to OA structural damage and progression, there is a paucity of longitudinal studies examining the relationship between imaging features and clinical symptoms [9,10,11,12,13]. Moreover, consistent correlations between clinical outcomes and imaging findings have not been found [14]. Clinical impairment constitutes a key determinant and indicator for medical or surgical treatment. Investigation of imaging biomarkers is important as they are closely related to symptom change, and longitudinal studies that correlate disease severity and progression with clinical outcomes are needed. The purpose of this study was to investigate whether OA-related MR imaging features correlate with longitudinal changes in clinical outcomes. The study population consisted of mildly to moderately symptomatic OA patients who received intra-articular injections over a 12-month period.

## 2. Materials and Methods

This retrospective study was conducted at a single academic medical center in accordance with the guidelines of the Declaration of Helsinki and received approval from the Institutional Review Board of Korea University Guro Hospital (2023GR0136). The requirement for informed consent was waived because of the retrospective nature of the study and the use of anonymized data.

### 2.1. Patients

The subjects were recruited from a group of knee OA patients who received intra-articular administration of 2 mL of hyaluronic acid (10 mg/mL; BMI Korea Co., Seoul, Republic of Korea) or 2 mL of sulfasalazine (2.4 mg/mL)-containing hyaluronic acid (10 mg/mL) (BMI Korea Co., Seoul, Republic of Korea) for one year. The patients underwent MRI and clinical assessment in evaluation of improvement or progression of OA at least twice, at baseline and after one year. Eligible participants were aged between 40 and 80 years and had medial femorotibial OA refractory to oral analgesics for at least 3 months, a baseline visual analog scale (VAS) score of 40 or higher, a baseline KL grade of 1–3 as determined by knee radiography, and a body mass index (BMI) between 18 and 35 kg/m^2^. Exclusion criteria were previous knee surgery or trauma, infection or inflammatory arthritis, and contraindications to MRI.

**Figure 1:**
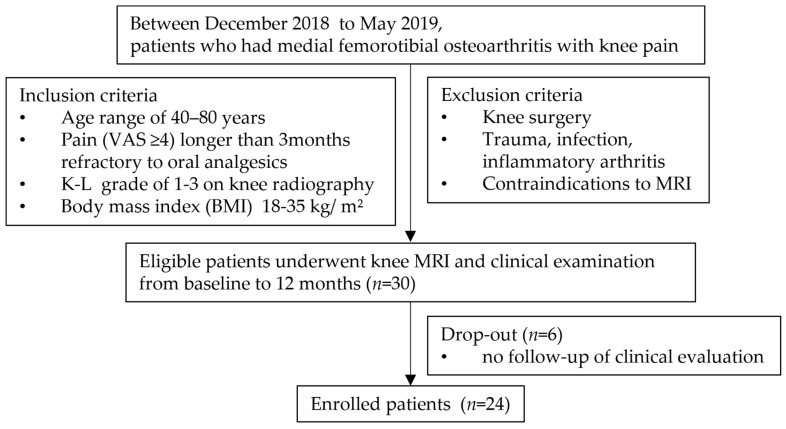
Flow chart illustrating patient selection.

Between December 2018 to May 2019, a total of 30 patients were initially enrolled in the study. Of these, six patients were excluded due to loss of follow-up, leaving 24 patients for the final retrospective analysis (Figure 1). The mean age of the participants was 67.00 ± 8.91 years, with a range from 42 to 79 years. The cohort consisted of 13 males (54%) and 11 females (46%). The mean BMI of the patients was 25.4 ± 3.2 kg/m^2^, with values ranging from 20.28 to 31.67 kg/m^2^. The distribution of KL grades at baseline was even, with eight patients each classified as KL grade I, II, and III. The baseline VAS score averaged 65.92 ± 10.60, reflecting moderate to severe pain levels among the participants.

### 2.2. Clinical Assessment

Clinical OA symptoms were assessed for 12 months from baseline using the VAS score, the Korean version of the Western Ontario and McMaster Universities Osteoarthritis Index (K-WOMAC) survey [15], and the Knee Injury and Osteoarthritis Outcome Score (KOOS) questionnaire [16]. The WOMAC survey is used to assess pain, stiffness, and physical function in OA patients. The possible score ranges are 0–20 for pain, 0–8 for stiffness, and 0–68 for physical function, with higher scores representing worse conditions. The KOOS survey evaluates the five categories of pain, symptoms, sport and recreation function, activities of daily living (ADL), and knee-related quality of life (QOL). The score ranges from 0 to 100, and a higher score is desirable. These clinical questionnaires were completed at baseline and at all follow-up visits by clinical staff.

### 2.3. Magnetic Resonance Imaging

All images were acquired using a 3T MR scanner (MAGNETOM Prisma, Siemens Healthcare, Erlangen, Germany) with a dedicated knee coil. Imaging sequences included sagittal three-dimensional (3D) fat-saturated (FS) proton density (PD)-weighted turbo spine echo (TSE) sampling perfection with application-optimized contrasts using different flip-angle evolution (SPACE), 3D true fast inflow with steady-state precession (FISP)-based T1ρ, and 2D multi-echo spin echo (MESE) T2 mapping sequences with the same position and FOV. The imaging parameters used in the study sequences are summarized in Table 1. The source data obtained from sagittal 3D FS PD-weighted images were subsequently reformatted into axial and coronal images. The 3D FS PD-weighted images were applied for semiquantitative assessment of knee joint OA severity and articular cartilage quantification. T1ρ and T2 mapping sequences were applied for quantitative compositional analysis of cartilage on a mid-sagittal image of the medial femoral condyle (MFC). All participants underwent MRI at baseline and 12 months post-baseline.

### 2.4. Image Analysis

#### 2.4.1. Semiquantitative Morphologic Assessment

**Figure 2:**
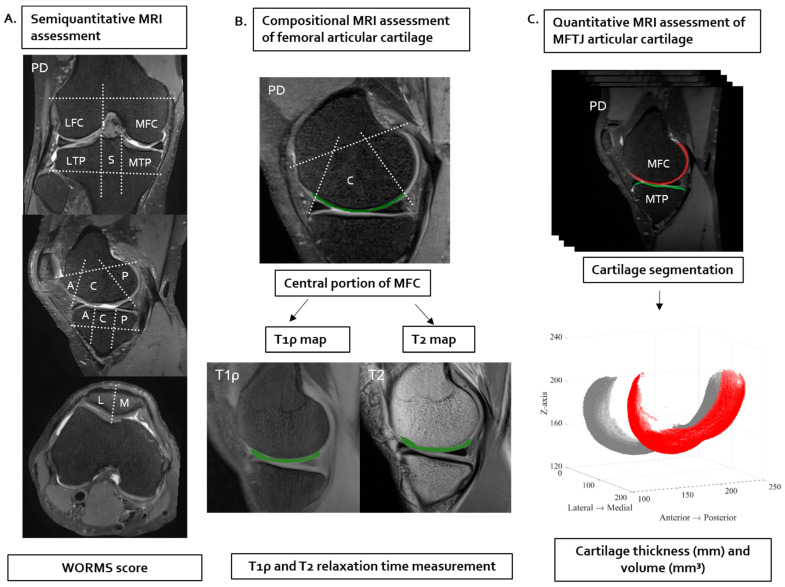
Illustration of methodology concerning MRI assessment. (**A**) Semiquantitative MRI whole joint assessment was performed on coronal, sagittal, and axial PD-weighted images using Whole-Organ Magnetic Resonance Imaging Score (WORMS). The lateral femoral condyle (LFC), medial femoral condyle (MFC), lateral tibial plateau (LTP), medial tibial plateau (MTP), and subspinous (S) regions are labeled (upper image). The femoral condyle and tibial plateau were each divided into three regions: anterior (A), central (C), and posterior (P), based on the anterior margin of the meniscus anterior horn and the posterior margin of the posterior horn (middle image). The patella was divided into the lateral facet (L) and medial facet (M) (lower image). (**B**) Compositional MRI assessment of articular cartilage was performed at the mid-sagittal plane of the MFC. The regions of interest (ROIs) in cartilage of central (C) and posterior (P) subregions were drawn manually on a PD-weighted image which provided the best anatomic contrast. The cartilage ROIs were then automatically copied and pasted onto the T1ρ and T2 maps to align positions. The automatically calculated T1ρ and T2 relaxation times (TRs) of each ROI were plotted in Microsoft Excel. (**C**) Quantitative MRI assessment of articular cartilage was performed at the medial femorotibial joint (MFTJ). The figure shows cartilage quantification of the MFC. Cartilage of MFTJ was segmented on each slice of PD-weighted images, and the thickness and volume of segmented cartilage were calculated using custom software (In-house software Ver 1.0). The red color indicates the cartilage of the medial femoral condyle, and the grey color indicates the cartilage of the lateral femoral condyle.

The baseline and 12-month follow-up 3D FS PD-weighted images were reviewed independently by two musculoskeletal radiologists with 19 and 9 years of experience, respectively, who were blinded to clinical information. Their reviews used a Whole-Organ Magnetic Resonance Imaging score (WORMS) system that combined 14 OA features from 15 sub-regions. The assessed features were articular cartilage integrity, subarticular bone marrow abnormality (BML), subarticular cysts, subarticular bone attrition, marginal osteophytes, medial and lateral meniscal integrity, anterior and posterior cruciate ligament integrity, medial and lateral collateral ligament integrity, synovitis/effusion, intraarticular loose bodies, and periarticular cysts/bursitis (Figure 2A).

#### 2.4.2. T1ρ and T2 Measurement

The sagittal 3D FS PD-weighted images were registered to match sagittal T1ρ and T2 images for accurate determination of the anatomic borders of cartilage (Figure 2B). The ROIs in the cartilage of central and posterior sub-regions on the mid-sagittal MFC image were drawn manually on a 3D FS PD-weighted image to obtain optimal anatomic contrast. The cartilage ROIs were automatically resampled and superimposed on the T1ρ and T2 maps to match the positioning. The T1ρ and T2 values of each ROI were automatically calculated and plotted in Microsoft Excel. Segmentation and measurements of T1ρ and T2 TRs were performed independently by two musculoskeletal radiologists. All image processing was performed using custom software developed in-house with MATLAB (Mathworks, Natick, MA, USA).

#### 2.4.3. Cartilage Thickness and Volume Quantitative Assessment

The sagittal 3D FS PD-weighted images at baseline and 12-month follow-up were used for cartilage segmentation (Figure 2C). The MFC and medial tibial plateau (MTP) cartilage were segmented separately using semiautomated segmentation software (In-house software Ver 1.0). The ROI mask in the cartilage compartment was drawn manually on each slice by two trained radiology technicians under the supervision of a musculoskeletal radiologist. Subsequently, the segmentation masks were transferred to a remote workstation and analyzed using custom software developed in-house using a pyKNEEr package for cartilage thickness and volume calculation [17].

### 2.5. Statistical Analysis

Descriptive statistics were calculated for all subjects. The Wilcoxon signed-rank test was performed to test for significant changes in MRI parameters (WORMS, T1ρ and T2 TRs, and cartilage thickness and volume) and clinical outcome measures (K-WOMAC and KOOS scores) between baseline and the 12-month follow-up. The associations between demographic data (age, BMI, and sex) and change in clinical status over 12 months were analyzed using Spearman’s correlation and Mann–Whitney test. The associations between imaging parameters and clinical outcome measures were assessed using Spearman’s correlation coefficients. These assessments included those of baseline imaging parameters, change in clinical outcome measures, and change in imaging parameters. The change between baseline and one-year follow-up was calculated by subtracting the baseline value from that of the one-year follow-up. Three patients were excluded when evaluating the association between T2 value and clinical outcome because of extreme outlier values (>200 ms) beyond the physiological range. These were most likely the result of misregistration or partial volume effect. Intraclass correlation coefficients (ICCs) were applied to determine inter-observer reliability. All analyses were performed using SPSS (version 25.0, IBM Corp., Armonk, NY, USA).

## 3. Results

The mean age of participants was 67.00 ± 8.91 years, approximately 54% were male, and the mean BMI was about 25 kg/m^2^. Eight patients each were classified as KL grades I, II, and III at baseline. The baseline VAS score was 65.92 ± 10.60. The baseline and 12-month follow-up clinical outcome scores and MRI parameters are presented in Table 2 and Table 3, respectively. There were no significant differences in changes from baseline for clinical outcome and MRI findings in the treatment group when using generalized estimating equation analyses.

Over the study period, clinical outcomes tended to improve. However, the improvement was statistically significant only for the mean KOOS symptom score (*p* = 0.004) (Table 2). Mean WORMS cartilage score for the medial femorotibial joint (MFTJ) and total joint were significantly increased (*p* = 0.31 and *p* = 0.00, respectively), and mean MTP cartilage thickness was significantly decreased (*p* = 0.03) (Table 3). There were no significant changes in other morphologic imaging features. The mean MFC T1ρ value showed a significant decrease in the central sub-region (*p* = 0.015).

### 3.1. Correlations between Demographic Factors and Changes in Clinical Outcome

Age was negatively correlated with changes in pain as measured by the K-WOMAC score (r = −0.413, *p* = 0.045). K-WOMAC function score was also negatively associated with age (r = −0.547, *p* = 0.006). Age was positively correlated with change in KOOS ADL score (r = 0.443, *p* = 0.030). BMI and sex had no significant relationship with clinical outcome changes.

### 3.2. Correlations of Changes in WORMS Scores, T1ρ and T2 Values, and Average Thickness and Volume of Cartilage with Changes in Clinical Outcome

**Figure 3:**
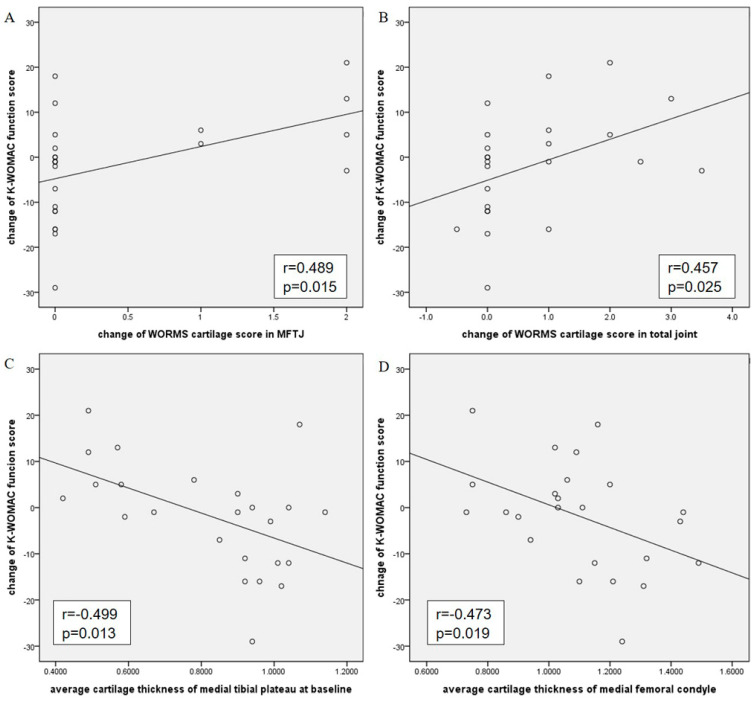
Correlations between change in Whole–Organ Magnetic Resonance Imaging Score (WORMS) score of medial femorotibial joint (MFTJ) cartilage (**A**) total joint, (**B**) average cartilage thickness of the medial tibial plateau (MTP), (**C**) medial femoral condyle (MFC), and (**D**) change in Korean Western Ontario and McMaster Universities (K-WOMAC) function score.

There was no significant correlation with change in clinical outcome among baseline WORMS parameters. However, over the 12-month study period, MFTJ and total joint cartilage score positively correlated with change in K-WOMAC function score (r = 0.489, *p* = 0.015; r = 0.457, *p* = 0.025) (Figure 3). Also, the change in WORMS total joint cartilage score negatively correlated with the change in KOOS pain score (r = −0.429, *p* = 0.036). Interestingly, the change in the WORMS cyst score of the patellofemoral joint (PFJ) was positively correlated with the change in KOOS pain score (r = 0.414, *p* = 0.044), and the change in the WORMS synovitis score was positively correlated with the change in the KOOS sport score (r = 0.439, *p* = 0.032). These findings suggest a complex relationship between structural changes and clinical symptoms. One possible explanation is that the formation of cysts and increased synovitis may reflect an underlying reparative or adaptive response within the joint. For instance, the development of cysts could be associated with the body’s attempt to redistribute synovial fluid to reduce pressure on certain joint areas, while synovitis might trigger an inflammatory response that initially exacerbates symptoms but later leads to tissue remodeling and symptom relief [18]. However, these hypotheses are speculative, and further research is needed to elucidate the precise molecular mechanisms underlying these observations.

The baseline T1ρ in the posterior MFC sub-region showed a positive correlation with the change in KOOS symptom score (r = 0.438, *p* = 0.037). No significant relationship was found between changes in T2 values and changes in clinical outcome measures.

The baseline average MTP cartilage thickness showed negative correlations with changes in K-WOMAC pain and function scores (r = −0.495, *p* = 0.014; r = −0.499, *p* = 0.013, respectively) (Figure 3) and positive correlations with changes in KOOS pain, symptom, and ADL and QOL scores (r = 0.420~0.526, *p* = 0.008~0.041). The baseline MTP cartilage volume also positively correlated with changes in KOOS pain and ADL scores (r = 0.449, *p* = 0.028; r = 0.490, *p* = 0.015, respectively). The baseline average MFC cartilage thickness negatively correlated with change in K-WOMAC function score (r = −0.473, *p* = 0.019) (Figure 3). These findings suggest that the presence of thicker and bulkier baseline cartilage was associated with clinical improvement. However, neither average MFTJ cartilage thickness nor cartilage volume changes correlated with changes in clinical outcome.

Interobserver agreement for WORMS parameters was, overall, moderate to excellent, except for bone attrition scores and WORMS parameters in the subspinous region. Interobserver agreement for bone attrition and WORMS parameters in the subspinous region were poor. Interobserver agreement for T2 values was excellent, and interobserver agreement for T1ρ values was moderate to good (Table 4).

## 4. Discussion

The interaction among clinical features, imaging findings, and outcome measures of OA development is complicated. In this longitudinal study, we assessed the evolution of joint pre-structural and structural features, cartilage volume and thickness, and cartilage biochemical composition as determined by knee MRI one year after initiation of intra-articular injection in patients with mildly to moderately symptomatic OA. Our findings indicate that quantitatively defined baseline MFTJ cartilage thickness and semiquantitatively assessed MFTJ and total joint cartilage loss over the 12 months are associated with changes in clinical outcome. These results confirm the important role of cartilage in knee OA progression.

MRI is a useful, non-invasive imaging tool for the evaluation of OA in longitudinal clinical trials. There have been conflicting reports regarding the association between MRI OA features and symptomatic progression [14]. Sayre et al. suggested that only the presence of osteophytes was significantly related to pain progression over a 7.5-year longitudinal early OA study period [19]. Magnusson et al. found that meniscal extrusion, full-thickness cartilage loss, and the presence of osteophytes were associated with increased knee pain in a six-year study of pre-radiographic OA patients [13]. Eckstein et al. demonstrated that reduced MFTJ cartilage thickness over two years had a strong association with radiographic progression but a weak association with pain progression [20]. In addition, some studies have demonstrated that increased severity of synovitis was related to increased pain severity [21]. However, our study showed that clinical improvement was significantly positively correlated with cyst progression and synovitis. These results imply that intra-articular injection provided some beneficial effects for advanced lesions in OA patients.

We found inconsistent results between quantitative and semiquantitative evaluations of cartilage. At baseline, quantitative measurements of cartilage thickness and volume correlated with changes in clinical outcome, but semiquantitative measurements did not. In contrast, when evaluating cartilage change over time, the semiquantitative method showed a significant correlation with changes in clinical outcome, but the quantitative method did not. However, previous studies have shown that quantitative measurement was more sensitive than semiquantitative scoring for the detection of cartilage morphology change [22,23]. In contrast, Reichenbach et al. reported that semiquantitative assessment could be more sensitive to detecting cartilage damage in mild OA because semiquantitative scores captured focal cartilage erosions that might have been missed by quantitative measurements that summarize morphology over a broad area [24]. We suggest that minor changes in the local area undetected by quantitative measurement can be captured by semiquantitative assessment.

T1ρ and T2 mapping methods are the most clinically applicable MRI techniques for evaluating biochemical composition before morphologic change. T1ρ mapping is believed to be sensitive in detecting early OA due to its inverse correlation with proteoglycan (PG) content, which depletes before collagen deterioration occurs, whereas T2 mapping is more related to collagen orientation and water content [25,26,27,28,29]. This suggests that T1ρ mapping may be more effective in identifying early PG depletion in OA. T1ρ may be more sensitive than T2 for early cartilage degeneration [30]. Interestingly, our analysis of the association between T1ρ and clinical outcome indicated a trend opposite to our expectation. Previous researchers provided incompatible findings that baseline T1ρ and T2 parameters are potential predictors of OA progression. The baseline MRI compositional markers may not provide better discrimination between knees with OA progression and those without significant progression than simple radiographic measures [31,32]. Edd et al. investigated the longitudinal changes in femoral cartilage T2 values and thickness in progressive OA and found that T2 values increased in the early stages, while cartilage thickness decreased primarily in the later stages [33]. These findings highlight the potential of cartilage imaging biomarkers to predict OA severity and progression depending on the disease stage. Similarly, a study by Li et al. demonstrated that T1ρ and T2 mapping sequences could identify early cartilage degeneration but were not effective in differentiating between the various Noyes classes of cartilage [34]. Moreover, T1ρ and T2 mappings showed comparable values in differentiating between moderate and severe OA, which aligns with our results. We hypothesize that the inclusion of patients with moderate OA in the target group may have influenced these outcomes.

The general belief is that joint symptoms as well as imaging findings of OA increase with age [35]. In this study, however, changes in clinical outcomes were found to be more favorable with increasing age. This may be explained by the relatively lower physical demands of older patients and higher expectations of younger patients. Also, previous studies demonstrated that patients over the age of 60 years with moderate OA are more likely to have a positive response to intra-articular HA administration [36].

There are several limitations in this study. First, our study included a small sample size and a heterogeneous population of KL grades from I to III. KL II and KL III knees, in particular, cover a wide spectrum of structural diseases that vary spatially [37]. The heterogeneity of KL II and III patients should be considered when selecting target subjects for clinical trials of disease progression. Therefore, further investigation using larger cohorts and homogeneous subject groups is needed to confirm our results. Second, we did not include the entire joint for quantitative analysis of cartilage; we only analyzed the MFTJ. Although OA is a disease involving the entire joint, the medial femorotibial compartment is the most sensitive region as medial regions experience greater loads that lead to greater cartilage loss [38]. Cartilage manual segmentation was time-consuming and labor-intensive and was performed only for the medial compartment. Third, the obtained T1ρ and T2 measurements may be inappropriate owing to contamination of the cartilage signal by synovial fluid. Mismatches related to different 2D and 3D image positions or knee movements may also affect these measurements. T1ρ and T2 values greater than 200 ms, more than double the highest expected mean T2 value, were considered outliers and were excluded from statistical analysis.

## 5. Conclusions

In conclusion, our study found that thicker baseline MFTJ cartilage and less progressive MFTJ and total joint cartilage loss were significantly positively correlated with clinical improvement over the 12-month study period in symptomatic knee OA patients following intra-articular injection. While our findings suggest that quantitative measures of cartilage thickness and volume can serve as sensitive morphological biomarkers of OA, the precision of MRI in assessing clinical outcomes remains uncertain. Given the variability in sub-regional cartilage changes, the semiquantitative assessment provided by MRI shows promise in detecting longitudinal changes. However, further research is needed to validate the accuracy and clinical relevance of MRI-based assessments for reliably predicting clinical outcomes in OA patients.

## Figures and Tables

**Table 1:** Knee MRI protocol acquisition parameters.

	Sagittal 3D, FS, PD-Weighted SPACE 3D	Sagittal 3D True FISP T1rho Mapping	Sagittal 2D MESE T2 Mapping
Repetition time (ms)	1000	6.3	4000.0
Echo time (ms)	45	3.1	13.0/26.0/39.0/52.0/65.0
Acquisition matrix	320 × 320	256 × 256	256 × 256
Field of view (mm)	160 × 160	160 × 160	160 × 160
Slice thickness (mm)	0.50	3.0	3.0
In-plane resolution (mm^2^)	0.5 × 0.5	0.6 × 0.6	0.6 × 0.6
Flip angle	120 (variable flip angle flag)	10	180
Parallel acquisition technique	CAIPIRINHA	-	-
Number of slices	240	160	160
Echo train length	38	0	5
Bandwidth per pixel (Hz)	390	400	225
Number of averages	1	1	1
Acquisition time	5 min 9 s	10 min 55 s	10 min 46 s

**Table 2:** Clinical outcome measures at baseline and 12-month follow-up.

		The Baseline	12-Month Follow-Up	*p*-Value
K-WOMAC	Pain	9.667 ± 3.226	8.875 ± 2.818	0.466
	Stiffness	3.917 ± 1.640	3.75 ± 1.359	0.748
	Function	33.792 ± 11.684	32 ± 10.384	0.582
KOOS	Pain	56.025 ± 14.923	60.424 ± 15.134	0.176
	Symptom	50.75 ± 12.376	61.013 ± 15.787	0.004
	ADL	62.363 ± 17.470	63.875 ± 17.224	0.789
	SPORT	42.292 ± 22.601	44.167 ± 23.344	0.789
	QOL	41.95 ± 21.285	41.95 ± 16.000	0.941

Data are means ± standard deviations.

**Table 3:** MRI features at baseline and 12-month follow-up.

		The Baseline	12-Month Follow-Up	*p*-Value
WORMS				
Medial femorotibial joint	Cartilage	12.5 (8, 18.25)	12.5 (9, 19.5)	0.031
	Bone marrow abnormality	1 (0, 2.5)	2 (0, 3)	n.s.
	Bone cysts	0.5 (0, 1)	1 (0, 1.5)	n.s.
	Bone attrition	0 (0, 0)	0 (0, 0)	-
	Osteophytes	3.5 (0, 12.5)	3.5 (0, 12.5)	-
	Meniscal lesion	4 (1, 5)	4 (1, 5)	n.s.
Lateral femorotibial joint	Cartilage	10.75 (4.5, 13.5)	10.75 (4.5, 13.5)	n.s.
	Bone marrow abnormality	0 (0, 1)	0 (0, 1)	n.s.
	Bone cysts	0 (0, 0)	0 (0, 0.5)	n.s.
	Bone attrition	0 (0, 0)	0 (0, 0)	-
	Osteophytes	2 (0, 7)	2 (0, 7)	n.s.
	Meniscal lesion	1 (0, 1.5)	1 (0, 1.5)	n.s.
Patellofemoral joint	Cartilage	8 (4.75, 11.5)	8 (5, 11.5)	n.s.
	Bone marrow abnormality	0 (0, 2)	0 (0, 1.5)	n.s.
	Bone cysts	1 (0, 1)	1 (0, 1.5)	n.s.
	Bone attrition	0 (0, 0.5)	0 (0, 0.5)	-
	Osteophytes	2 (0, 9.5)	2 (0, 9.5)	-
Subspinous region	Bone marrow abnormality	0 (0, 1)	0 (0, 1)	n.s.
	Bone cysts	0.5 (0, 1)	1 (0, 1)	n.s.
Total joint	Cartilage	32 (20.5, 46.25)	32.75 (21, 46.75)	0.002
	Bone marrow abnormality	3 (0, 6)	2.5 (0.5, 5)	n.s.
	Ligament lesions	1.75 (1, 3)	1.75 (1, 3)	n.s.
	Synovitis	1 (1, 2)	1 (1, 2)	n.s.
T1ρ and T2 TRs in the medial femoral condyle				
T1ρ (ms)	Central	46.208 (40.542, 60.085)	41.744 (38.988, 46.809)	0.015
	Posterior	45.407 (41.294, 51.195)	47.372 (41.806, 33.994)	n.s.
T2 (ms)	Central	70.595 (56.062, 74.092)	68.773 (56.683, 84.347)	n.s.
	Posterior	53.937 (47.567, 70.850)	51.476 (45.866, 58.958)	n.s.
Average cartilage thickness and cartilage volume				
Medial femoral condyle	Average cartilage thickness (mm)	1.095 (0.98, 1.225)	1.065 (0.99, 1.245)	n.s.
	Volume (mm^3^)	2043 (1726.5, 2660.5)	2173.5 (1869.5, 2530.5)	n.s.
Medial tibial plateau	Average cartilage thickness (mm)	0.91 (0.585, 1)	0.83 (0.58, 0.89)	0.033
	Volume (mm^3^)	1119.5 (911.5, 1345.5)	1107 (926.5, 1322.5)	n.s.

Data are median and interquartile range (IQR). n.s. stands for “not significant”.

**Table 4:** Interobserver agreements for Whole-Organ Magnetic Resonance Imaging Score (WORMS) and T1 ρ and T2 relaxation times.

		ICC
WORMS		
Medial femorotibial joint	Cartilage	0.948
	Bone marrow abnormality	0.883
	Bone cysts	0.774
	Bone attrition	0.456
	Osteophytes	0.931
	Meniscal lesion	0.923
Lateral femorotibial joint	Cartilage	0.895
	Bone marrow abnormality	0.906
	Bone cysts	0.677
	Bone attrition	0.011
	Osteophytes	0.738
	Meniscal lesion	0.864
Patellofemoral joint	Cartilage	0.850
	Bone marrow abnormality	0.809
	Bone cysts	0.766
	Bone attrition	0.688
	Osteophytes	0.894
Subspinous region	Bone marrow abnormality	0.368
	Bone cysts	0.317
Total joint	Ligament lesions	0.601
	Synovitis	0.501
T1ρ and T2 TRs in the medial femoral condyle		
T1ρ	Central	0.757
	Posterior	0.652
T2	Central	0.971
	Posterior	0.950

Interobserver agreements are expressed as intraclass correlation coefficients (ICCs).

## Data Availability

Data supporting the present study are available from the corresponding author upon reasonable request.
